# Third Trimester Equivalent Alcohol Exposure Reduces Modulation of Glutamatergic Synaptic Transmission by 5-HT_1A_ Receptors in the Rat Hippocampal CA3 Region

**DOI:** 10.3389/fnins.2016.00266

**Published:** 2016-06-08

**Authors:** Russell A. Morton, C. Fernando Valenzuela

**Affiliations:** Department of Neurosciences, School of Medicine, University of New Mexico Health Sciences CenterAlbuquerque, NM, USA

**Keywords:** fetal alcohol spectrum disorders, fetal alcohol syndrome, serotonin, hippocampus, synaptic transmission, rat model

## Abstract

Fetal alcohol exposure has been associated with many neuropsychiatric disorders that have been linked to altered serotonin (5-hydroxytryptamine; 5-HT) signaling, including depression and anxiety. During the first 2 weeks of postnatal life in rodents (equivalent to the third trimester of human pregnancy) 5-HT neurons undergo significant functional maturation and their axons reach target regions in the forebrain (e.g., cortex and hippocampus). The objective of this study was to identify the effects of third trimester ethanol (EtOH) exposure on hippocampal 5-HT signaling. Using EtOH vapor inhalation chambers, we exposed rat pups to EtOH for 4 h/day from postnatal day (P) 2 to P12. The average serum EtOH concentration in the pups was 0.13 ± 0.04 g/dl (legal intoxication limit in humans = 0.08 g/dl). We used brain slices to assess the modulatory actions of 5-HT on field excitatory postsynaptic potentials in the hippocampal CA3 region at P13-P15. Application of the GABA_A_/glycine receptor antagonist, picrotoxin, caused broadening of field excitatory postsynaptic potentials (fEPSPs), an effect that was reversed by application of 5-HT in slices from air exposed rats. However, this effect of 5-HT was absent in EtOH exposed animals. In slices from naïve animals, application of a 5-HT_1A_ receptor antagonist blocked the effect of 5-HT on the fEPSPs recorded in presence of picrotoxin, suggesting that third trimester ethanol exposure acts by inhibiting the function of these receptors. Studies indicate that 5-HT_1A_ receptors play a critical role in the development of hippocampal circuits. Therefore, inhibition of these receptors by third trimester ethanol exposure could contribute to the pathophysiology of fetal alcohol spectrum disorders.

## Introduction

The hippocampal formation plays a central role in learning and memory processes (Ranganath, 2010; Buzsaki and Moser, 2013). A critical period for hippocampal development is the third trimester of human pregnancy (equivalent to the first 1–2 weeks of postnatal life in rodents). The number of synapses dramatically increases during this period and spontaneous activity drives the formation and early refinement of hippocampal neuronal networks (Mohajerani and Cherubini, 2006; Lohmann and Kessels, 2014). In addition, there is an increase in the levels of serotonin (5-hydroxytryptamine; 5-HT), which promotes dendritic maturation and regulates brain-derived neurotrophic factor levels in the developing hippocampus (Gaspar et al., 2003; Frederick and Stanwood, 2009; Migliarini et al., 2013). The elevation in 5-HT levels is a consequence of a dramatic increase in innervation of the hippocampus by serotoninergic axonal terminals originating in the dorsal raphe nucleus (Lidov and Molliver, 1982). Moreover, 5-HT neurons in the developing dorsal raphe nucleus undergo significant functional changes during the third trimester equivalent (Rood et al., 2014; Morton et al., 2015). Therefore, the 5-HT neurotransmitter system is actively refined during this developmental period, making it potentially susceptible to a number of insults.

Among the factors that can have an impact on the 5-HT neurotransmitter system is fetal ethanol exposure, a leading cause of intellectual disability across the globe (Murawski et al., 2015; Roozen et al., 2016). Studies with humans have demonstrated that prenatal ethanol exposure alters brain stem 5-HT neurons, which may explain the increased association between sudden infant death syndrome and fetal alcohol spectrum disorder (FASD; Kinney et al., 2003). Kraemer et al. (2008) found that prenatal ethanol-exposed monkeys carrying a 5-HT transporter gene polymorphism were more irritable as neonates and exhibited larger neuroendocrine responses to stress. Studies performed with rodents have shown that exposure to ethanol during prenatal development reduces both the number of 5-HT neurons in the dorsal raphe nucleus as well as the projections of these neurons (Druse et al., 2004; Sari and Zhou, 2004; Zhou et al., 2005; Sliwowska et al., 2014). Moreover, Weinberg and colleagues demonstrated that prenatal ethanol exposure persistently alters the function of 5-HT_1A_ and 5-HT_2A∕C_ receptors in a sex-dependent manner (Hofmann et al., 2002, 2005, 2007; Sliwowska et al., 2008). However, little is known about the effect of ethanol exposure during the third trimester equivalent on the 5-HT system.

Alterations in the actions of 5-HT during the third trimester equivalent have been shown to have long-lasting behavioral consequences. Depletion of 5-HT in postnatal day (P)10-P20 rats caused spatial learning deficits during adolescence, which was associated with dendritic alterations in the hippocampus (Mazer et al., 1997). Perinatal exposure of rodents to selective 5-HT reuptake inhibitors increased anxiety behavior during adulthood via alterations in the function of brain-derived neurotrophic factor (Ansorge et al., 2004; Noorlander et al., 2008; Karpova et al., 2009; Boulle et al., 2015). Studies suggest that these effects are, in part, mediated by enhanced activity of 5-HT_1A_ receptors, which are expressed both in neuronal and glial cells during this critical period of hippocampal development (Borella et al., 1997; Patel and Zhou, 2005). 5-HT_1A_ receptor activation at P6 increased cell division in the dentate gyrus and strengthened synaptic transmission in the CA1 region of cultured mouse hippocampal slices, an effect that depended on activation of extracellular signal regulated kinases 1 and 2 and protein kinase C (Mehta et al., 2007). Injection of a 5-HT_1A_ agonist between P5 and P14 prevented the loss of dentate granule cell dendritic spines induced by 5-HT depletion, whereas injection of an antagonist of this receptor caused comparable dendritic spine loss to that produced by 5-HT depletion (Yan et al., 1997). Enhanced activity of 5-HT_1A_ receptors during a portion of the third trimester equivalent increased the formation of dendritic spines and synapses in the murine hippocampus (Mogha et al., 2012). Collectively, these findings indicate that disruption of the 5-HT neurotransmitter system during the third trimester equivalent can alter the developmental trajectory of hippocampal circuits.

In this study, we investigated the impact of ethanol exposure during the third trimester equivalent on the modulatory actions of 5-HT in CA3 hippocampal region. We focused on this region because it has been shown to be an important target of the developmental actions of ethanol (West et al., 1981; Savage and Reyes, 1985; West and Hamre, 1985; Maier and West, 2001; Livy et al., 2003; Galindo et al., 2005; Mameli et al., 2005; Zucca and Valenzuela, 2010). We found evidence consistent with inhibition of 5-HT_1A_ receptor function in the CA3 hippocampal region of ethanol-exposed rats.

## Materials and methods

Animal procedures were approved by the University of New Mexico Health Sciences Center Institutional Care and Use Committee. All chemicals were from Sigma-Aldrich (St. Louis, MO) or Tocris (Bristol, U.K.). Timed-pregnant Sprague-Dawley rats (Harlan Laboratories, Indianapolis, IN) arrived at our animal facility between gestational days 12 and 15. Rats were randomly assigned to the control and ethanol groups. We exposed pups and dams to ethanol for 4 h/day between P2 and 12 using vapor inhalation chambers that were constructed as previously described (Morton et al., 2014). Control rats were exposed only to air in the same type of chambers. For some control experiments, we used naïve pups that were neither exposed to air nor ethanol. Serum ethanol levels were assessed in pups randomly selected throughout the exposure paradigm at the end of the 4 h period of exposure. Pups were anesthetized with isoflurane (Piramal Healthcare, Andhra Pradesh, India) followed by decapitation. Fifty microliter of trunk blood was collected, mixed with 6.6% perchloric acid (450 μL), centrifuged for 15 min at 1600 g, and the supernatant was stored at −80°C in sealed tubes until ready to use. Blood ethanol levels were determined using an alcohol dehydrogenase-based assay, as previously reported (Galindo and Valenzuela, 2006). Brain slices were prepared at P13–15 by heavily anesthetizing animals with 0.75 g/kg ketamine followed by decapitation. Brain tissue was removed and immediately incubated for 2–4 min in oxygenated ice-cold cutting solution containing (in mM): KCl, 2; NaH_2_PO_4_, 1.3; NaHCO_3_, 26; MgSO_4_, 12; CaCl_2_, 0.2; sucrose, 220; glucose, 10; ketamine hydrochloride, 1 μg/mL. Coronal brain slices of 250 μm thickness were generated using a vibrating slicer (1000 Plus Vibratome, Leica, Bannockburn, Illinois). Slices were incubated in oxygenated artificial cerebral spinal fluid (ACSF) at 35°C for 40 min followed by storage at 21–22°C for at least 30 min prior to recording. The ACSF contained (in mM): NaCl, 125; KCl, 2; NaH_2_PO_4_, 1.3; NaCO_3_, 26; glucose, 10, CaCl_2_, 2; MgSO_4_, 1. Recordings were performed at 32°C. Slices were visualized with an Olympus BX51WI upright microscope (Olympus, Center Valley, PA) using a Plan 4x lens 0.1 N.A (Olympus) and a complementary metal-oxide semiconductor digital camera (Q-Imaging, Surrey, Canada). Patch pipettes (2–3 MΩ) were pulled from thin wall filament-containing borosilicate capillary glass using a Sutter Flaming-Brown P-97 multi-stage puller (Sutter Instruments. Novato, California) and were filled with ACSF. Recordings were performed with a Multiclamp 700B amplifier and a Digidata 1440A interface; pClamp 9 software was used for data acquisition (Molecular Devices Sunnyvale, CA). Since pups were at an early developmental stage (equivalent to a human baby) at the time of electrophysiological recordings (P13–15), we did not discriminate between male and female animals and pooled data for both sexes. Statistical analyses were carried out using Prism 5.0 (GraphPad Software, San Diego, CA). The unit of determination was defined as an animal (data generated from slices from a single animal were averaged). All data sets were tested for outliers using the Rout test with Q = 1%.

## Results

**Figure 1:**
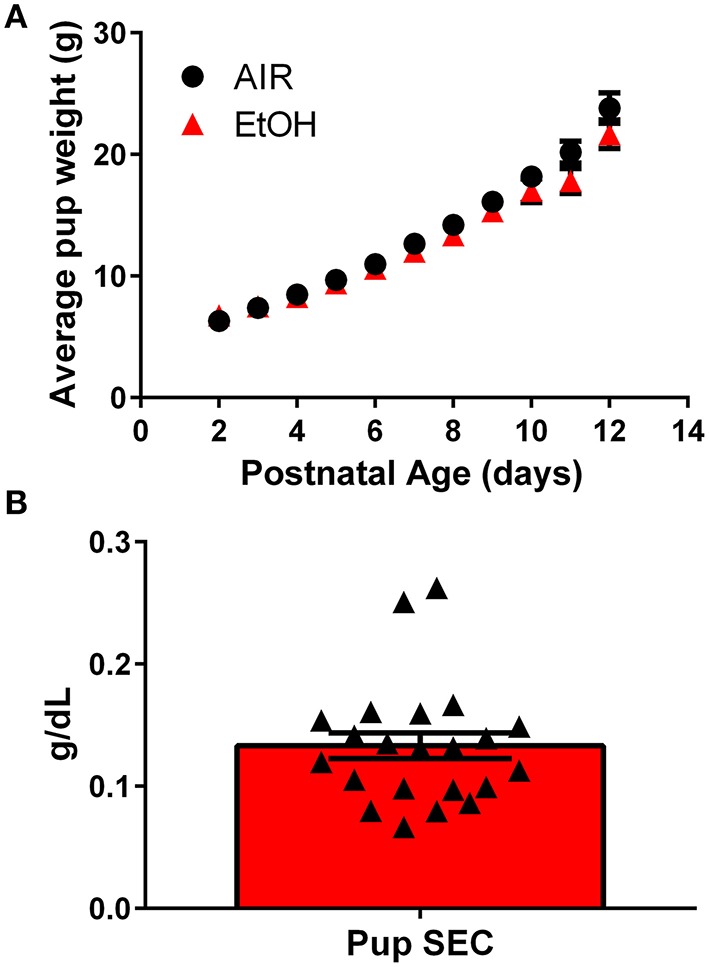
**Characterization of the third trimester-equivalent ethanol exposure paradigm: pup weight gain and serum ethanol levels. (A)** Average pup weight for air and ethanol (EtOH) exposed animals. **(B)** Averaged pup serum EtOH concentration measured at the end of the 4 h-long exposure (*n* = 22 animals from 10 litters). Triangles represent values obtained in individual pups.

We used a vapor inhalation paradigm to expose pups housed with their mothers to ethanol during the third trimester-equivalent period. Pups were exposed to ethanol vapor for 4 h each day from P2 to 12 and experiments were conducted at P13–15. Third trimester-equivalent ethanol exposure did not affect pup weight (Figure 1A; two-way ANOVA; interaction, *F*_(10, 250)_ = 3.231, *P* = 0.0006; postnatal days, *F*_(10, 250)_ = 358.8, *P* < 0.001; exposure, *F*_(1, 25)_ = 1.375, *P* = 0.2521). The average serum ethanol concentrations were 0.13 ± 0.05 g/dL (~ 31 mM; Figure 1B).

**Figure 2:**
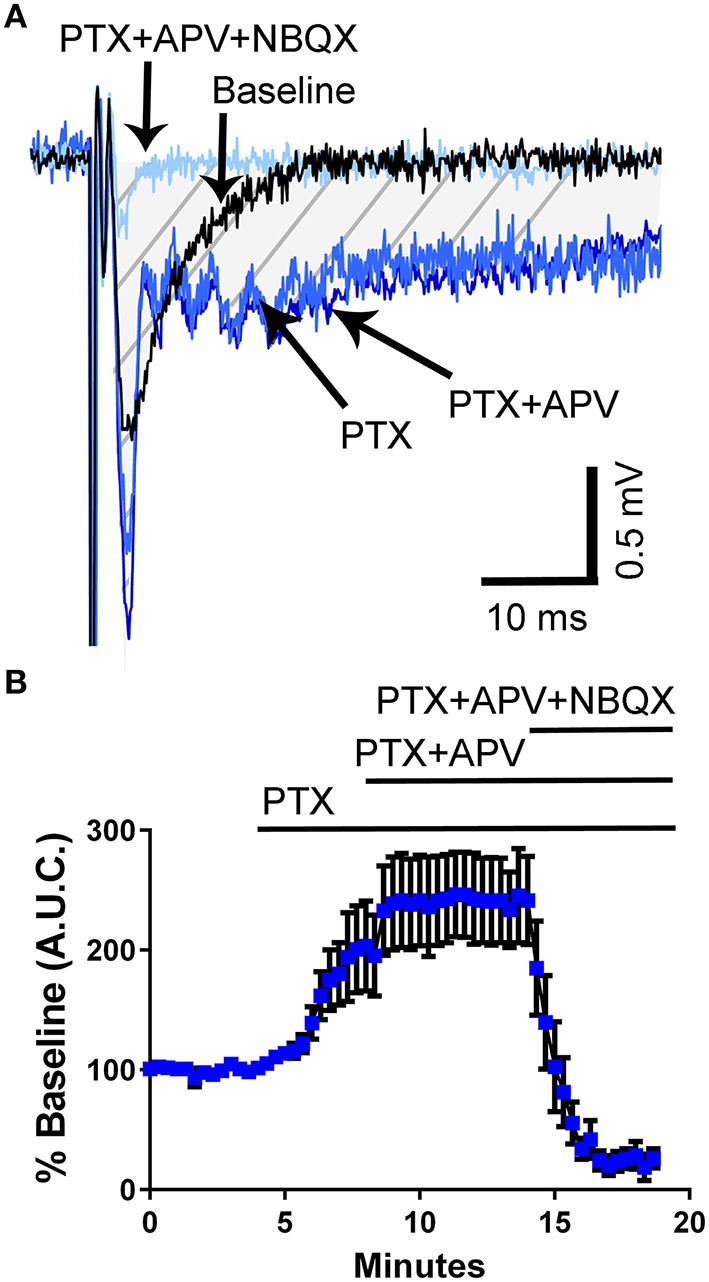
**Application of the GABA_A_/glycine receptor antagonist, picrotoxin, induces broadening of field excitatory postsynaptic potentials in the CA3 hippocampal region from un-exposed naïve animals. (A)** Sample traces illustrating the effect of picrotoxin (PTX; 50 μM) and the lack of effect of the NMDA receptor antagonist, APV (100 μM). The field excitatory post-synaptic potentials were blocked by the non-NMDA receptor antagonist, NBQX (10 μM). The shaded hatched area indicates where we measured area under the curve (A.U.C.). **(B)** Summary of results obtained with eight slices from three pups from 2 litters (see text for results of statistical analyses).

Studies have shown that 5-HT can reverse epileptiform activity in hippocampal neurons (Salgado-Commissariat and Alkadhi, 1996; Lu and Gean, 1998; Tokarski et al., 2002; Thone and Wiemann, 2007). To determine if third trimester-equivalent ethanol exposure affects this effect of 5-HT, we evoked local field excitatory postsynaptic potentials (fEPSPs) via electrical stimulation with a concentric bipolar electrode placed in the *stratum lucidum* near the CA3 pyramidal cell layer to stimulate CA3-to-CA3 synapses. We then exposed the slices to the GABA_A_/glycine receptor antagonist, picrotoxin (PTX), which resulted in widening of the fEPSP (Figures 2A,B). We quantified the area under the curve (A.U.C.) represented by the marked area in the representative traces in Figure 2A. The application of PTX significantly increased the fEPSP A.U.C. (repeated measures one-way ANOVA *F*_(3, 21)_ = 21.67, *P* = 0.0001; Holm-Sidak's *post-hoc* test = *P* < 0.05 at 2–4 vs. 6–8 min); however, addition of the NMDA receptor antagonist, APV (100 μM) did not have a significant effect on the fEPSP recorded in PTX (Holm-Sidak's *post-hoc* test *P* > 0.05 at 6–8 vs. 12–14 min; Figures 2A,B), suggesting that PTX does not broaden the fEPSP by activating NMDA receptors via membrane potential depolarization and removal of Mg^2+^ block. Application of the non-NMDA receptor antagonist, NBQX (10 μM), abolished the fEPSP (Figures 2A,B).

**Figure 3:**
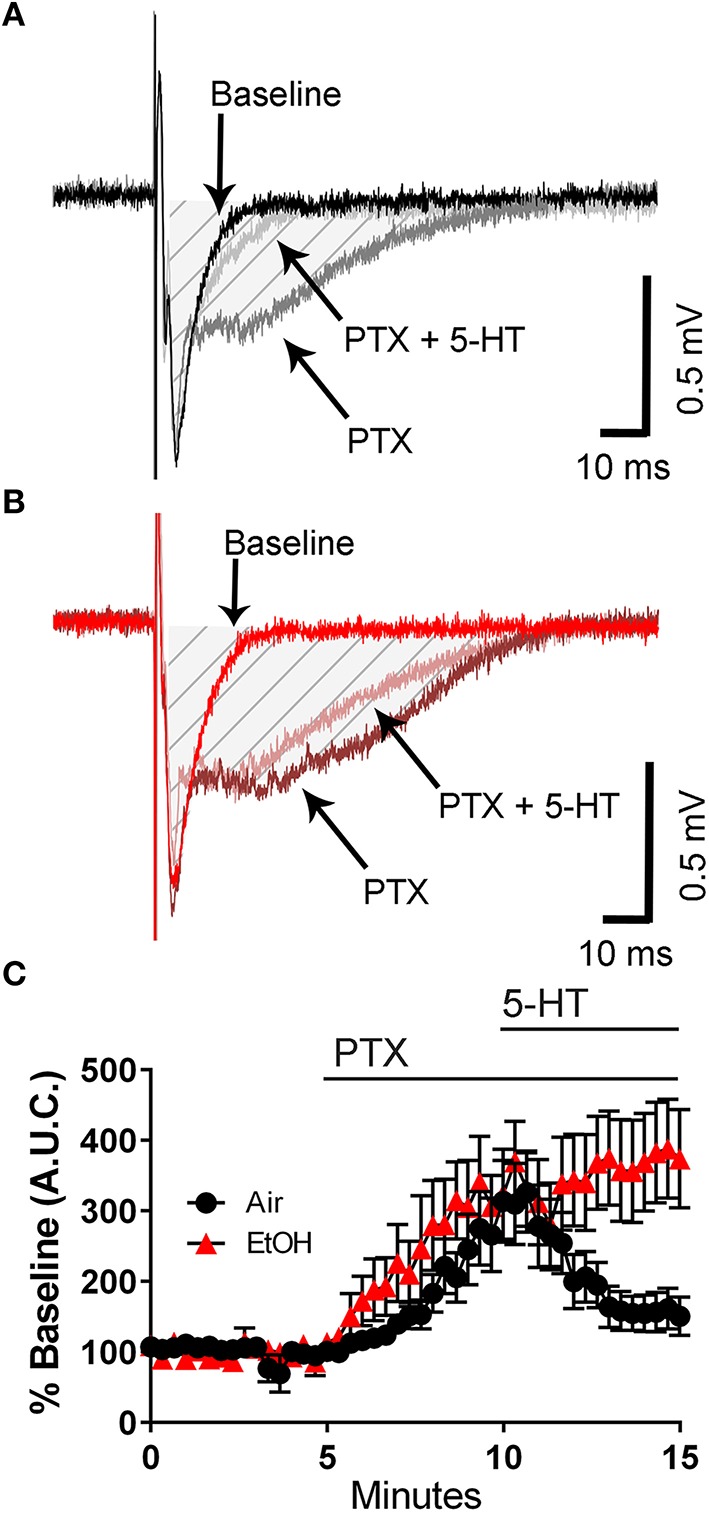
**Third trimester-equivalent ethanol (EtOH) exposure blocks the 5-HT-induced inhibition of field excitatory post-synaptic potentials recorded under conditions of GABA_A_/glycine receptor inhibition**. Representative traces from air **(A)** and EtOH **(B)** exposed animals at baseline, in the presence of 50 μM picrotoxin (PTX), and in the presence of 50 μM PTX and 100 μM 5-HT. The shaded hatched area indicates where we measured area under the curve (A.U.C.). **(C)** Time course of the normalized A.U.C.s in all three conditions. See text for results of statistical analyses (*n* = 9–10 animals from 5 litters).

We next tested the effect of 5-HT on the PTX-induced broadening of the fEPSP. Although PTX increased the fEPSP A.U.C. to a similar extent in slices from control and ethanol-exposed rats, the ability of 5-HT to reduce the fEPSP A.U.C. was significantly impaired in slices from ethanol-exposed rats (Figures 3A–C; two-way ANOVA: interaction, *F*_(45, 782)_ = 1.912, *P* = 0.0004; time, *F*_(45, 782)_ = 9.481, *P* < 0.0001; exposure, *F*_(1, 782)_ = 66.15, *P* < 0.0001; Tukey's *post-hoc* test = *P* < 0.05 at 10–11 min vs. baseline for air and 9–15 min vs. baseline for ethanol; Sidak's *post-hoc* test = *P* < 0.05 air vs. ethanol at 13–15 min). These data suggest that third trimester-equivalent ethanol exposure significantly blunts 5-HT signaling in the CA3 region.

**Figure 4:**
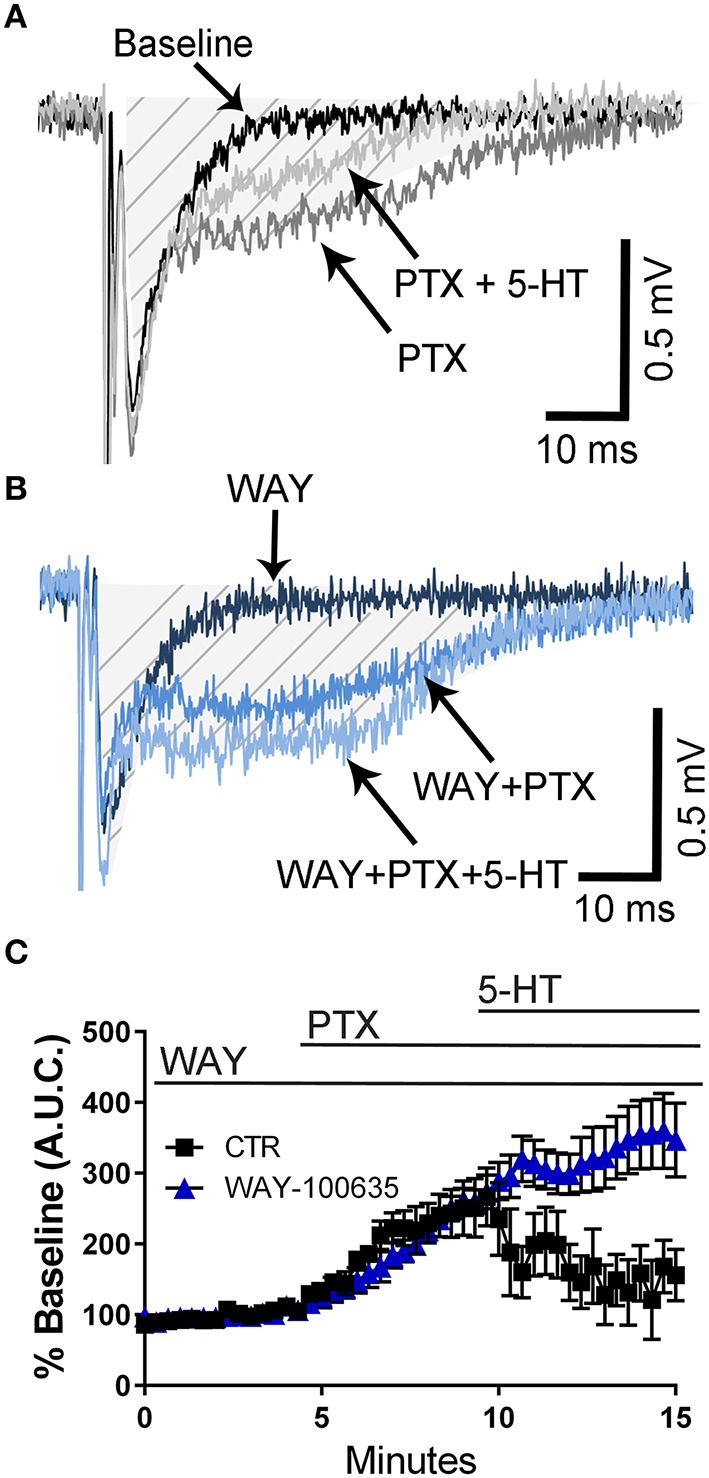
**Pharmacological blockade of 5-HT_1A_ receptors blocks the 5-HT-induced inhibition of field excitatory post-synaptic potentials recorded under conditions of GABA_A_/glycine receptor inhibition in slices from naïve rats. (A)** Representative traces from a naïve animal obtained at baseline, in the presence of 50 μM picrotoxin (PTX), and in the presence of 50 μM PTX + 100 μM 5-HT. **(B)** Same as in panel A but in the constant presence of the 5-HT_1A_ antagonist, WAY-100635 (10 μM). The shaded hatched area indicates where we measured area under the curve (A.U.C.). **(C)** Time course of the effect of 5-HT on the PTX-induced increase of the AUC in absence and presence of WAY-100635. See text for results of statistical analyses (*n* = 6 animals from 2 litters).

It has been demonstrated that 5-HT reduces epileptiform activity in rat hippocampal CA1 neurons via activation of 5-HT_1A_ receptors (Salgado-Commissariat and Alkadhi, 1996; Lu and Gean, 1998). Consequently, we investigated whether these receptors could mediate the effect of 5-HT on fEPSPs recorded in presence of PTX. In naïve animals, not exposed to air or ethanol, application of the selective 5-HT_1A_ receptor antagonist, WAY- 100635 did not affect the PTX-induced increase of the fEPSP A.U.C. (Figures 4A–C). However, this agent blocked the effect of 5-HT on the fEPSP A.U.C. (Figures 4A–C) (two-way ANOVA: interaction, *F*_(45, 460)_ = 4.325, *P* < 0.0001; time, *F*_(45, 460)_ = 11.02, *P* < 0.0001; WAY-100635, *F*_(1, 460)_ = 70.58, *P* < 0.0001; Tukey's *post-hoc* test = *P* < 0.05 at 9–10 min vs. baseline for air and 9–15 min vs. baseline for ethanol; Sidak's *post-hoc* test = *P* < 0.05 air vs. ethanol at 10.6 and 12–15 min).

## Discussion

We report here a novel mechanism of action of ethanol during brain development. We found that exposure of rats during a period of development equivalent to the third trimester of human pregnancy significantly blunts the modulatory effects of 5-HT on fEPSPs recorded in the CA3 hippocampal region under conditions of GABA_A_/glycine receptor blockade. Blockade of 5-HT_1A_ receptors with the antagonist WAY100636 mimicked the effect of ethanol, suggesting that these receptors mediate the inhibitory effect of 5-HT. These findings are in agreement with studies showing that 5-HT can reverse epileptiform activity in the CA1 region of mature rats and guinea pigs through 5-HT_1A_ receptor activation (Salgado-Commissariat and Alkadhi, 1996; Lu and Gean, 1998; Tokarski et al., 2002; Thone and Wiemann, 2007). They are also consistent with results of a previous study demonstrating that activation of 5-HT_1A_ receptors inhibits excitatory postsynaptic potentials to a greater extent in the presence than in the absence of the GABA_A_ receptor antagonist, bicuculine (Pugliese et al., 1998). Activation of 5-HT_1A_ receptors has been shown to inhibit glutamate release in several neuronal populations and this effect could be, in part, responsible for the decrease the fEPSP A.U.C. (Cheng et al., 1998; Torres-Escalante et al., 2004; Costa et al., 2012; Choi et al., 2013). It has also been demonstrated that these receptors inhibit AMPA receptor function via inhibition of calcium/calmodulin-dependent protein kinase II activity, leading to dephosphorylation of the GluA1 subunit by protein phosphatase 1 (Cai et al., 2002; Schiapparelli et al., 2006; Costa et al., 2012). Therefore, inhibition of postsynaptic AMPA receptors by 5-HT_1A_ receptors could also play a role in the reduction of the fEPSP A.U.C. by 5-HT. It is well-established that activation of 5-HT_1A_ receptors induces membrane potential hyperpolarization in CA1 and CA3 pyramidal neurons by activating somato-dendritic inward rectifier K^+^ channels and inhibiting the hyperpolarization-activated current (Haddjeri and Blier, 1995; Sodickson and Bean, 1998; Gasparini and Difrancesco, 1999; Bickmeyer et al., 2002; Tokarski et al., 2002). This effect can also contribute to the 5-HT-induced reduction of the fEPSP A.U.C. via a decrease in glutamate release at CA3-to-CA3 synapses and/or a shunting mechanism.

Chronic exposure of rats to ethanol during pregnancy (equivalent to the first and second trimesters of human pregnancy) has been shown to delay the developmental increase in 5-HT_1A_ receptor levels that normally takes place in the cerebral cortex and lateral septum between postnatal days 19 and 35; this effect could be prevented by treatment with agonists of these receptors, suggesting that prenatal ethanol exposure inhibits 5-HT_1A_ receptor function (Tajuddin and Druse, 1988; Kim et al., 1997; Druse et al., 2004, 2005, 2006). It is possible that third trimester-equivalent ethanol exposure also caused a reduction in 5-HT_1A_ receptor levels, explaining the lack of a 5-HT effect on fEPSP A.U.C. This effect could be a consequence of reduced production or increased degradation of these receptors. Alternatively, third trimester ethanol exposure could cause uncoupling of the receptors from the G protein or alterations in components of the downstream signaling pathways activated by 5-HT_1A_ receptors. It is important to experimentally address these possibilities in the future. Studies from the Weinberg laboratory have demonstrated that chronic prenatal ethanol exposure can actually produce long-lasting alterations in both the levels and the function of 5-HT_1A_ receptors in a sex-dependent manner (Hofmann et al., 2005, 2007; Sliwowska et al., 2008). Therefore, it should also be investigated whether the third trimester ethanol exposure-induced inhibition of 5-HT_1A_ receptor function persist into adolescence and adulthood, whether it also occurs in other neuronal populations, and if the effect is sex dependent.

In summary, our data suggest that 5-HT signaling in the hippocampal CA3 region is impaired by third trimester ethanol exposure. Furthermore, our data suggest that these effects of third trimester ethanol exposure may be due to impaired function of 5-HT_1A_ receptors. Knockout mice for 5-HT_1A_ receptors are more anxious and conditional rescue of expression of these receptors in the hippocampus and cortex (but not the raphe nuclei) restores normal behavior in these mice (Heisler et al., 1998; Parks et al., 1998; Ramboz et al., 1998; Gross et al., 2002). More recently, it was shown that blockade of 5-HT_1A_ receptors during the early postnatal period induces a persistent increase in anxiety-like behavior in mice (Vinkers et al., 2010). Consequently, future studies should investigate whether third trimester ethanol exposure-induced alterations of 5-HT_1A_ receptor function increase susceptibility to mood disorders later in life.

## Author contributions

RM designed and performed the experiments, analyzed data, and wrote the manuscript. CV assisted with experimental design and data analyses, supervised the project, and wrote the manuscript.

### Conflict of interest statement

The authors declare that the research was conducted in the absence of any commercial or financial relationships that could be construed as a potential conflict of interest.
